# Hypothermic Ventricular Fibrillation in Redo Minimally Invasive Mitral Valve Surgery: A Promising Solution for a Surgical Challenge

**DOI:** 10.3390/jcm13144269

**Published:** 2024-07-22

**Authors:** Jawad Salman, Maximilian Franz, Khalil Aburahma, Nunzio Davide de Manna, Saleh Tavil, Sadeq Ali-Hasan-Al-Saegh, Fabio Ius, Dietmar Boethig, Alina Zubarevich, Bastian Schmack, Tim Kaufeld, Aron-Frederik Popov, Arjang Ruhparwar, Alexander Weymann

**Affiliations:** Department of Cardiothoracic, Transplantation and Vascular Surgery, Hannover Medical School, 30625 Hannover, Germany; salman.jawad@mh-hannover.de (J.S.); schmack.bastian@mh-hannover.de (B.S.); weymann.alexander@mh-hannover.de (A.W.)

**Keywords:** redo minimally invasive mitral valve surgery, hypothermic ventricular fibrillation, outcomes

## Abstract

**Background**: Minimally invasive mitral valve surgery (MIMVS) is a treatment for severe mitral valve pathologies. In redo cases, especially after coronary artery bypass grafting (CABG) surgery with patent mammary bypass grafts, establishing aortic clamping followed by antegrade cardioplegia application might be challenging. Here, we present the outcome of hypothermic ventricular fibrillation as an alternative to conventional cardioprotection. **Methods**: Patients who underwent MIMVS either received hypothermic ventricular fibrillation (study group, *n* = 48) or antegrade cardioprotection (control group, *n* = 840) and were observed for 30 postoperative days. Data were retrospectively analyzed and collected from January 2011 until December 2022. **Results**: Patients in the study group had a higher preoperative prevalence of renal insufficiency (*p* = 0.001), extracardiac arteriopathy (*p* = 0.001), insulin-dependent diabetes mellitus (*p* = 0.001) and chronic lung disease (*p* = 0.036). Furthermore, they had a longer surgery time and a lower repair rate (*p* < 0.001). No difference, however, was seen in postoperative incidences of stroke (*p* = 0.26), myocardial infarction (*p* = 1) and mitral valve re-operation (*p* = 1) as well as 30-day mortality (*p* = 0.1) and postoperative mitral valve insufficiency or stenosis. **Conclusions**: The patients who underwent redo MIMVS with hypothermic ventricular fibrillation did not have worse outcomes or more serious adverse events compared to the patients who received routine conventional cardioprotection. Therefore, the use of hypothermic ventricular fibrillation appears to be a promising cardioprotective technique in this challenging patient population requiring redo MIMVS.

## 1. Introduction

Minimally invasive mitral valve surgery (MIMVS) has emerged as the treatment for severe mitral valve pathologies [1]. The development of MIMVS has been driven by advancements in surgical instrumentation, imaging modalities, and procedural approaches. Cardiac surgeons have refined and optimized techniques such as port-access, robot-assisted, and endoscopic-guided mitral valve interventions [1,2,3]. These minimally invasive methods have allowed for smaller incisions, reduced surgical trauma, and improved patient outcomes. Numerous studies have proven the feasibility and excellent outcomes of MIMVS when compared to traditional mitral valve surgery with full sternotomy [1,2,3].

Redo MIMVS represents a specialized and technically challenging surgical approach for patients requiring repeat interventions on the mitral valve. This technique is particularly beneficial for individuals who have previously undergone open-heart procedures, as it aims to mitigate the complexities and risks associated with repeat sternotomies [1,4,5,6]. In redo cases, especially after CABG surgery with patent mammary artery bypass grafts, it can be challenging to clamp the mammary bypass and establish aortic clamping followed by antegrade cardioprotection [2,7,8,9]. In situations where safely mobilizing the ascending aorta is not feasible, the use of an endoclamp can facilitate the surgical procedure. Alternatively, hypothermic ventricular fibrillation may serve as a viable option, as it allows the surgeon to operate on a protected heart while keeping the ascending aorta untouched [10,11].

In this study, we retrospectively analyzed the early postoperative outcomes of hypothermic ventricular fibrillation in redo minimally invasive mitral valve surgery.

## 2. Materials and Methods

### 2.1. Study Population and Design

Between January 2011 and December 2022, a total of 888 minimally invasive mitral valve surgeries were performed at our center. Among them, 48 patients received hypothermic ventricular fibrillation (study group), and 840 patients underwent antegrade cardioprotection (control group). Peri- and postoperative data were collected from our prospectively maintained database. Patient follow-up was conducted until the 30th postoperative day.

### 2.2. Ethical Statement

In accordance with local German protocols, study approval by the institutional ethical review board was waived given the retrospective and non-interventional design of this study.

### 2.3. Surgical Technique

The surgical technique has already been described elsewhere [12,13,14]. To sum up, single-lung ventilation was used, and cardiopulmonary bypass was usually established via the right inguinal vessels. First, a venous two-stage cannula was inserted over the right femoral vein into the superior vena cava under echocardiographic control. Then, the arterial cannula was inserted. All surgeries were performed via a right minithoracotomy. Carbon dioxide was insufflated during the whole procedure. The pericardium was opened 3–4 cm above the phrenic nerve. Hypothermia at 30 °C was induced by cardiopulmonary bypass which resulted in ventricular fibrillation. If this did not work, we also used electric shocks to induce fibrillation. Cerebral blood oxygen concentration was measured. Unfractionated heparin was intravenously applied to elevate the activated clotting time above 450 s, and after the removal of the venous cannula, antagonization with protamine was carried out. Postoperative transthoracic echocardiography was performed routinely before discharge and mitral valve function was evaluated. Stenosis was graded according to Omran et al. [15]. Insufficiency was graded as described by Chew et al. [16].

### 2.4. Outcomes Measures

The primary endpoint was 30-day mortality. Postoperative early mortality was defined as death occurring within the hospital stay and the first 30 days after operation. Secondary endpoints were the duration of postoperative intensive care unit (ICU) stay, as well as the incidence of postoperative ischemic stroke, right ventricular failure, new-onset atrial fibrillation, new-onset myocardial infarction, the need for pacemaker implantation, major bleeding requiring re-thoracotomy, sepsis, and renal failure requiring dialysis. Preoperative renal failure was attributed to patients who were diagnosed with acute or chronic renal failure by the referring hospital according to generally accepted parameters (decreased urine production, glomerular filtration rate).

### 2.5. Variables and Definitions

Variables were evaluated, including patient characteristics, further preoperative clinical assessments, laboratory parameters before surgery, intraoperative data, postoperative variables, and follow-up data. Elective, urgent, and emergency operations were performed, and the outcomes were compared between the groups. Elective patients were admitted routinely. Urgent patients were not admitted electively and needed to be operated on during the same hospital stay without the option to be sent home or already showed signs of heart failure. Emergency patients received surgery the same day the decision for surgery was made.

### 2.6. Echocardiographic Assessment

Preoperative and postoperative echocardiographic characteristics were analyzed, i.e., the preoperative left ventricular ejection fraction (LVEF), the rate of mitral valve insufficiency (MI) II, MI III and MI IV, as well as the rate of mitral valve stenosis (MS) II and MS III. The same characteristics were also analyzed postoperatively before hospital discharge.

### 2.7. Subgroup-Analysis

To explore the exact comparison, a sub-analysis was performed between the study group and the 7% of patients in the control group who had a history of cardiac surgery. The results are shown in the Supplementary Materials (Tables S1–S4).

### 2.8. Statistical Analysis

The data analysis was conducted using SPSS statistical software, version 28.01.1. For continuous variables, the results were summarized using medians and interquartile ranges. Categorical variables were described as the number of cases and the corresponding percentage relative to the overall study population. To compare continuous variables between groups, the Mann–Whitney U test was utilized. For categorical variables, Chi-square testing was employed. If any cell in the crosstab analysis had an expected count less than 5, Fisher’s exact test was performed instead. A *p*-value less than 0.05 was considered statistically significant throughout the analyses.

## 3. Results

### 3.1. Baseline Characteristics

Patients from the study group had a median age of 70 years and patients from the control group had a median age of 67 years. Of all participants, 65% in the study group and 55% in the control group were male. All patients in the study group had undergone previous cardiac surgery (100%), compared to only 7% in the control group. The rate of preoperative CABG in the study group was 75%. One of the patients in the study group underwent transcatheter aortic valve replacement (TAVR), and another had a porcelain aorta, which is associated with a high risk of stroke during aortic clamping.

The distribution of pre-operative heart failure severity, as measured by New York Heart Association (NYHA) class, was similar between the two groups, with NYHA III being the most common in both groups. The timing of the procedures, whether elective, urgent, or emergency, was also comparable between the groups (*p* = 0.09). Patients in the study group had a higher prevalence of various comorbidities, including preoperative renal failure, extracardiac arteriopathy, chronic obstructive lung disease, insulin-dependent diabetes mellitus, pulmonary hypertension, coronary artery disease, arterial hypertension, hyperlipidemia, and atrial fibrillation.

Mitral valve regurgitation was the most frequent operative indication in both the study group (90%) and the control group (86%) (*p* = 0.67). However, the study group had a lower incidence of Carpentier type I (4% vs. 12%, *p* < 0.001) and Carpentier type II (21% vs. 56%, *p* < 0.001) mitral valve lesions. In contrast, they more often suffered from Carpentier type III lesions (42% vs. 17%; *p* < 0.001) (Table 1).

### 3.2. Intraoperative Characteristics

The study group had a significantly longer overall surgical duration, with a median time of 235 min compared to 205 min in the control group (*p* < 0.001). However, the time spent on cardiopulmonary bypass was similar between the two groups, with a median of 154 min in the study group and 137 min in the control group (*p* = 0.27). The study group had a higher rate of biological valve replacement (54% vs. 24%, *p* < 0.001) and mechanical valve replacement (31% vs. 11%, *p* < 0.001) compared to the control group. Consequently, the mitral valve repair rate was lower in the study group (15% vs. 65%, *p* < 0.001), as were the rates of mitral valve plasty with neochordae (15% vs. 46%, *p* < 0.001) and cleft closure (0% vs. 9%, *p* = 0.017) (Table 2).

### 3.3. Postoperative Characteristics

The time of postoperative catecholamine therapy (22 h vs. 17 h; *p* < 0.001), postoperative ventilation time (14 h vs. 11 h; *p* < 0.001), and time in the intensive care unit (4 days vs. 1 day; *p* = 0.001) were longer in the study group (Table 3). However, there were no significant differences between the groups in the rates of several postoperative outcomes, including the need for mitral valve re-operation (0% vs. 2%, *p* = 1); arrhythmias in general (21% vs. 11%, *p* = 0.067), including new-onset atrial fibrillation (10% vs. 10%, *p* = 0.99); pneumothorax (6% vs. 5%, *p* = 0.74); right ventricular failure requiring extracorporeal membrane oxygenation (ECMO) implantation (8% vs. 3%, *p* = 0.084); stroke (4% vs. 2%, *p* = 0.26); and cerebral bleeding (2% vs. 0%, *p* = 0.26). In contrast, the study group had a higher risk of postoperative complications, including a greater need for re-thoracotomy due to major bleeding (21% vs. 7%, *p* = 0.07) and pacemaker implantation (14% vs. 5%, *p* = 0.016). The study group also had a higher incidence of postoperative renal insufficiency requiring dialysis (13% vs. 3%, *p* = 0.003), although only 6% of the study group required ongoing dialysis after hospital discharge. The in-hospital mortality (6% vs. 2%, *p* = 0.1) and 30-day mortality (6% vs. 2%, *p* = 0.1) rates were similar between the two groups (Table 3). Out of the three deceased patients from the study group, one died due to sepsis, the second due to atherosclerotic mesenteric ischemia, and the third after suffering from postoperative low cardiac output syndrome. The first measured Creatine Kinase-MB (CK-MB) concentration was 61 (52–84) U/L in the study group and 51 (39–70) U/L in the control group. The lactate concentration was 1.6 (1.275–2.875) mmol/L in the study group and 1.4 (1–2) in the control group.

### 3.4. Echocardiographic Assessments

The preoperative echocardiographic assessment revealed that the study group had a lower left ventricular ejection fraction compared to the control group (56% vs. 60%, *p* = 0.012). However, the two groups were comparable in all other echocardiographic parameters evaluated, as shown in Table 4.

## 4. Discussion

This retrospective study analyzed the early postoperative outcomes of redo MIMVS, where hypothermic ventricular fibrillation was used as cardioprotection. The study found that hypothermic ventricular fibrillation did not negatively impact 30-day mortality rates or the incidence of postoperative stroke and cerebral bleeding. However, our study did find a higher rate of postoperative renal failure requiring dialysis in the study group. It is important to note that these patients also had a higher prevalence of preexisting renal failure preoperatively. Furthermore, only half of the patients who required new onset dialysis postoperatively were under dialysis after hospital discharge. Additionally, the study group showed a higher rate of postoperative pacemaker implantation, which can be explained by the higher prevalence of preoperative atrial fibrillation in this group. Despite the high-risk profile of the patients in our study, the causes of death were primarily non-cardiac in nature.

Milani et al. analyzed ten patients who received MIMVS as reoperation with hypothermic ventricular fibrillation. Their results showed a 0% early postoperative mortality rate in this patient group [17]. According to our findings, the early postoperative mortality rate was 6%. However, two out of three deceased patients died due to mesenteric ischemia and sepsis, which were unrelated to cardioprotection. The third patient died due to postoperative low cardiac output syndrome. This patient was 75 years old and had preexisting NYHA Class IV heart failure. After the urgent mitral valve replacement procedure, the patient required veno-arterial (VA)-ECMO support for two postoperative days.

Romano et al. also investigated patients who underwent MIMVS with ventricular fibrillation as redo cardiac surgery. Their results showed a mean cardiopulmonary bypass time of 113 min, a mean postoperative ventilation time of 34 h, and a 30-day mortality rate of 7.4%. The stroke rate was 2.9%, and the incidences of sepsis, hemothorax, and renal failure requiring hemodialysis were 2.9%, 1.5%, and 1.5%, respectively [18]. In comparison with our findings, the current study’s results showed a median cardiopulmonary bypass time of 154 min, a median postoperative ventilation time of 14 h, and a 30-day mortality rate of 6%. The stroke rate was 4%, and the incidences of sepsis, hemothorax, and renal failure requiring dialysis were 0%, 21%, and 13%, respectively. However, only 6% of patients were still on dialysis after hospital discharge. To sum up, 30-day mortality was comparable, although we had a higher rate of postoperative bleeding and requirement for postoperative new-onset dialysis.

A study by Davierwala et al. reported a 30-day mortality rate of only 0.8% after MIMVS. However, their cohort had a much lower rate of prior cardiac surgery at 5.4%. In contrast, the current study’s patient population had a much higher rate of prior cardiac procedures, with 96% being cardiac redo cases [19]. Despite this, the 30-day mortality rate in our database was 6%, which is comparable to the 6.6% rate reported in the US STS database for mitral valve surgery after previous cardiac procedures [20]. Importantly, none of the deceased patients in the current study died due to the surgical procedure itself. The stroke rate of 4% in our study was also comparable to the 2% rate reported by Davierwala et al. [19]. However, the incidence of re-thoracotomy due to postoperative bleeding was higher in our study at 21%, compared to 7% in the Davierwala et al. study [19].

It should be noted that 75% of our patients who received MIMVS under ventricular fibrillation had undergone CABG with one of the bypasses being the left internal mammary artery to left anterior descending artery (LIMA-LAD), 17% had undergone previous valve replacement and one patient (4%) had previously received closure of an atrial septum defect. The difference in terms of previous cardiac procedures explains the seemingly higher complication rate of our study group. Additionally, a high incidence of preoperative renal failure (46% of our study group) naturally resulted in a higher demand for postoperative dialysis. Patients who underwent redo cardiac surgery were frequently under platelet inhibition or anticoagulation and, most importantly, had intrathoracic adhesions, all factors that tremendously increased the postoperative bleeding risk.

Hypothermic ventricular fibrillation in MIMVS provides a safe technique for high-risk patients who had already undergone cardiac surgery, such as CABG or valve replacement. In an aging population, it is a promising approach given the increasing number of patients who will undergo redo cardiac surgery. It also allows for a minimally invasive approach, preventing conversion to sternotomy, which is known to result in a longer postoperative hospital stay and is unpopular among patients [4].

One of the primary benefits of utilizing a right mini-thoracotomy approach for redo mitral valve surgery is the ability to avoid the risks associated with sternal re-entry and dissection of adhesions. This minimally invasive technique helps limit the potential for injury to cardiac structures or patent bypass grafts while also reducing the amount of postoperative bleeding [8]. Additionally, performing mitral valve surgery under ventricular fibrillation can help prevent the risk of systemic embolization that may occur with the use of aortic clamping, especially in patients with severe aortic calcification [21,22,23]. Current knowledge and our single-center experience suggest that MIMVS under hypothermic ventricular fibrillation without aortic cross-clamping through a right minithoracotomy is a safe, reproducible and effective option for patients requiring redo mitral valve surgery, especially when the patient has anatomical characteristics that increase the risk of re-sternotomy such as coronary bypass grafts.

## 5. Conclusions

The patients who underwent redo MIMVS with hypothermic ventricular fibrillation did not have worse outcomes or more serious adverse events compared to the patients who received routine conventional cardioprotection. Therefore, the use of hypothermic ventricular fibrillation appears to be a promising cardioprotective technique in this challenging patient population requiring redo MIMVS. The study has several limitations, including that the analysis was not performed using propensity score matching. In addition, the data only included the early follow-up period. To address these limitations, further studies with a larger patient cohort and long-term follow-up are required.

## Figures and Tables

**Table 1:** Preoperative characteristics.

Variables	Study Group (*N* = 48)	Control Group (*N* = 840)	*p*-Value
Age	70 (65–76)	67 (56–75)	0.015
Male Gender	31 (65%)	459 (55%)	0.15
NYHA I	0 (0%)	36 (4%)	0.42
NYHA II	14 (29%)	326 (39%)	
NYHA III	27 (56%)	343 (41%)	
NYHA IV	2 (4%)	38 (5%)	
Elective operations	25 (52%)	547 (65%)	0.092
Urgent operations	17 (35%)	237 (28%)	
Emergency operations	6 (13%)	52 (6%)	
Cardiac re-operation	48 (100%)	60 (7%)	<0.001
Preoperative renal failure	22 (46%)	133 (16%)	<0.001
Extracardiac arteriopathy	13 (27%)	51 (6%)	<0.001
Chronic obstructive lung disease	13 (27%)	122 (15%)	0.036
Active endocarditis	2 (4%)	57 (7%)	0.75
Insulin dependent diabetes mellitus	11 (23%)	22 (3%)	<0.001
Recent myocardial infarction	1 (2%)	12 (1%)	0.52
Preoperative stroke	3 (6%)	69 (8%)	0.79
Preoperative neurologic symptoms	6 (13%)	88 (10%)	0.63
Pulmonary hypertension	32 (67%)	368 (44%)	0.003
Coronary artery disease	36 (75%)	217 (26%)	<0.001
Smoking history	12 (25%)	184 (22%)	0.60
Family history	3 (6%)	65 (8%)	1
Arterial hypertension	45 (94%)	555 (66%)	<0.001
Hyperlipidemia	40 (83%)	372 (44%)	<0.001
Atrial fibrillation	29 (60%)	376 (45%)	0.038
Mitral valve stenosis	3 (6%)	50 (6%)	0.76
Mitral valve regurgitation	43 (90%)	723 (86%)	0.67
Endocarditis	2 (4%)	41 (8%)	0.42
Mitral valve prolapse	14 (29%)	506 (60%)	<0.001
Mitral chord rupture	8 (17%)	363 (43%)	<0.001
Anulus dilatation	28 (38%)	418 (50%)	0.18
Carpentier I	2 (4%)	97 (12%)	<0.001
Carpentier II	10 (21%)	470 (56%)	<0.001
Carpentier III	20 (42%)	124 (17%)	<0.001

Continuous variables were described as the median and interquartile range. Categorical variables were described as mean with the related percentage.

**Table 2:** Intraoperative data.

Variables	Study Group (*N* = 48)	Control Group (*N* = 840)	*p*-Value
Surgery time (min)	235 (200–265)	205 (176–240)	<0.001
Time on CPB (min)	154 (228–178)	137 (114–165)	0.27
Mitral valve replacement			
Biological	26 (54%)	201 (24%)	<0.001
Mechanical	15 (31%)	90 (11%)	<0.001
Mitral valve repair	7 (15%)	549 (65%)	<0.001
Plasty with neochordae	7 (15%)	390 (46%)	<0.001
Number of neochordae ^§^	0 (0–0)	0 (0–2)	<0.001
Cleft closure	0 (0%)	82 (9%)	0.017
Segment resection	1 (2%)	40 (5%)	0.72
Sliding plasty	0 (0%)	5 (1%)	1
Augmentation	0 (0%)	33 (4%)	0.25
LAA closure	6 (13%)	246 (29%)	0.013
Maze procedure	3 (6%)	166 (20%)	0.021

CPB: cardiopulmonary bypass; LAA: left atrial appendage. Continuous variables were described as median and interquartile range. Categorical variables were described as number with the related percentage. **^§^** Data presented as medians and interquartile ranges.

**Table 3:** Postoperative data.

Variables	Study Group (*N* = 48)	Control Group (*N* = 840)	*p*-Value
Catecholamine duration	22 (17–68)	17 (8–27)	<0.001
Ventilation time (h)	14 (10–33)	11 (7–16)	<0.001
ICU stay (days)	4 (1–6)	1 (1–3)	0.001
Mitral valve re-operation	0 (0%)	13 (2%)	1
Arrhythmia	10 (21%)	96 (11%)	0.067
Pneumothorax	3 (6%)	44 (5%)	0.74
ECMO/right ventricular failure	4 (8%)	27 (3%)	0.084
Re-thoracotomy due to Bleeding	10 (21%)	57 (7%)	0.01
New atrial fibrillation	5 (10%)	82 (10%)	0.99
Renal insufficiency with new onset dialysis	6 (13%)	23 (3%)	0.003
Stroke	2 (4%)	16 (2%)	0.26
Cerebral bleeding	1 (2%)	2 (0%)	0.15
Peripheral vascular complications	0 (0%)	5 (0%)	1
Sepsis	0 (0%)	12 (1%)	0.52
Myocardial infarction	0 (0%)	2 (0%)	1
Pacemaker implantation	7 (14%)	43 (5%)	0.016
30-day mortality	3 (6%)	18 (2%)	0.10
In-hospital mortality	3 (6%)	18 (2%)	0.10

ICU: intensive care unit; ECMO: extracorporeal membrane oxygenation. Continuous variables were described as median and interquartile range. Categorical variables were described as mean with the related percentage.

**Table 4:** Echocardiographic measurements.

Time	Variables	Study Group (N = 48)	Control Group (N = 840)	*p*-Value
Preoperative	LVEF	56 (44–62)	60 (53–65)	0.012
	MI II	11 (23%)	140 (17%)	0.92
	MI III	30 (63%)	563 (67%)	0.70
	MI IV	5 (10%)	78 (9%)	0.79
	MS II	1 (2%)	13 (2%)	0.53
	MS III	0 (0%)	23 (3%)	0.31
Postoperative	LVEF	55 (44–60)	53 (45–60)	0.74
	MI I	7 (15%)	155 (18%)	0.57
	MI II	0 (0%)	19 (2%)	0.62
	MI III	0 (0%)	1 (3%)	-
	MI IV	0 (0%)	0 (0%)	-
	MS II	0 (0%)	0 (0%)	-
	MS III	0 (0%)	0 (0%)	-

Continuous variables were described as median and interquartile range. Categorical variables were described as number with the related percentage.

## Data Availability

Data are contained within the article and Supplementary Materials.

## Supplementary Materials

The following supporting information can be downloaded at: https://www.mdpi.com/article/10.3390/jcm13144269/s1, Table S1: Preoperative characteristics; Table S2: Intraoperative data; Table S3: Post-operative data; Table S4: Echocardiographic measurements

## References

[1] Akowuah E.F., Maier R.H., Hancock H.C., Kharatikoopaei E., Vale L., Fernandez-Garcia C., Ogundimu E., Wagnild J., Mathias A., Walmsley Z. (2023). Minithoracotomy vs. Conventional Sternotomy for Mitral Valve Repair: A Randomized Clinical Trial. JAMA.

[2] Güllü A., Şenay Ş., Ersin E., Demirhisar Ö., Whitham T., Koçyiğit M., Alhan C. (2021). Robotic-assisted cardiac surgery without aortic cross-clamping: A safe alternative approach. J. Card. Surg..

[3] Kofler M., Van Praet K.M., Schambach J., Akansel S., Sündermann S., Schönrath F., Jacobs S., Falk V., Kempfert J. (2021). Minimally invasive surgery versus sternotomy in native mitral valve endocarditis: A matched comparison. Eur. J. Cardiothorac. Surg..

[4] Eqbal A.J., Gupta S., Basha A., Qiu Y., Wu N., Rega F., Chu F.V., Belley-Cote E.P., Whitlock R.P. (2022). Minimally invasive mitral valve surgery versus conventional sternotomy mitral valve surgery: A systematic review and meta-analysis of 119 studies. J. Card. Surg..

[5] Santana O., Larrauri-Reyes M., Zamora C., Mihos C.G. (2016). Is a minimally invasive approach for mitral valve surgery more cost-effective than median sternotomy?. Interact. Cardiovasc. Thorac. Surg..

[6] Kastengren M., Svenarud P., Källner G., Franco-Cereceda A., Liska J., Gran I., Dalén M. (2020). Minimally invasive versus sternotomy mitral valve surgery when initiating a minimally invasive programme. Eur. J. Cardiothorac. Surg..

[7] Barbero C., Costamagna A., Verbrugghe P., Zacharias J., Van Praet F., Bove T., Agnino A., Kempfert J., Rinaldi M. (2024). Clinical Impact of the Endo-aortic Clamp for Redo Mitral Valve Surgery. J. Cardiovasc. Transl. Res..

[8] Botta L., Cannata A., Bruschi G., Fratto P., Taglieri C., Russo C.F., Martinelli L. (2013). Minimally invasive approach for redo mitral valve surgery. J. Thorac. Dis..

[9] Zwischenberger B.A., Gaca J.G., Milano C., Carr K., Glower D.D. (2024). Late Survival After Redo Mitral Operation with Minithoracotomy Compared with Sternotomy. Ann. Thorac. Surg..

[10] Prestipino F., D’Ascoli R., Nagy Á., Paternoster G., Manzan E., Luzi G. (2021). Mini-thoracotomy in redo mitral valve surgery: Safety and efficacy of a standardized procedure. J. Thorac. Dis..

[11] Murzi M., Miceli A., Di Stefano G., Cerillo A.G., Farneti P., Solinas M., Glauber M. (2014). Minimally invasive right thoracotomy approach for mitral valve surgery in patients with previous sternotomy: A single institution experience with 173 patients. J. Thorac. Cardiovasc. Surg..

[12] Fleissner F., Salman J., Naqizadah J., Avsar M., Meier J., Warnecke G., Kuhn C., Cebotari S., Ziesing S., Haverich A. (2019). Minimally Invasive Surgery in Mitral Valve Endocarditis. Thorac. Cardiovasc. Surg..

[13] Franz M., De Manna N.D., Schulz S., Ius F., Haverich A., Cebotari S., Tudorache I., Salman J. (2023). Minimally Invasive Mitral Valve Surgery in the Elderly. Thorac. Cardiovasc. Surg..

[14] Salman J., Fleissner F., Naqizadah J., Avsar M., Shrestha M., Warnecke G., Ismail I., Rumke S., Cebotari S., Haverich A. (2018). Minimally Invasive Mitral Valve Surgery in Re-Do Cases-The New Standard Procedure?. Thorac. Cardiovasc. Surg..

[15] Omran A.S., Arifi A.A., Mohamed A.A. (2011). Echocardiography in mitral stenosis. J. Saudi Heart Assoc..

[16] Chew P.G., Bounford K., Plein S., Schlosshan D., Greenwood J.P. (2018). Multimodality imaging for the quantitative assessment of mitral regurgitation. Quant. Imaging Med. Surg..

[17] Milani R., Brofman P.R., Oliveira S., Patrial Neto L., Rosa M., Lima V.H., Binder L.F., Sanches A. (2013). Minimally invasive redo mitral valve surgery without aortic crossclamp. Rev. Bras. Cir. Cardiovasc..

[18] Romano M.A., Haft J.W., Pagani F.D., Bolling S.F. (2012). Beating heart surgery via right thoracotomy for reoperative mitral valve surgery: A safe and effective operative alternative. J. Thorac. Cardiovasc. Surg..

[19] Davierwala P.M., Seeburger J., Pfannmueller B., Garbade J., Misfeld M., Borger M.A., Mohr F.W. (2013). Minimally invasive mitral valve surgery: “The Leipzig experience”. Ann. Cardiothorac. Surg..

[20] Kilic A., Acker M.A., Gleason T.G., Sultan I., Vemulapalli S., Thibault D., Ailawadi G., Badhwar V., Thourani V., Kilic A. (2019). Clinical Outcomes of Mitral Valve Reoperations in the United States: An Analysis of The Society of Thoracic Surgeons National Database. Ann. Thorac. Surg..

[21] Crooke G.A., Schwartz C.F., Ribakove G.H., Ursomanno P., Gogoladze G., Culliford A.T., Galloway A.C., Grossi E.A. (2010). Retrograde arterial perfusion, not incision location, significantly increases the risk of stroke in reoperative mitral valve procedures. Ann. Thorac. Surg..

[22] Svensson L.G., Blackstone E.H., Rajeswaran J., Sabik J.F., Lytle B.W., Gonzalez-Stawinski G., Varvitsiotis P., Banbury M.K., McCarthy P.M., Pettersson G.B. (2004). Does the arterial cannulation site for circulatory arrest influence stroke risk?. Ann. Thorac. Surg..

[23] Etz C.D., Plestis K.A., Kari F.A., Silovitz D., Bodian C.A., Spielvogel D., Griepp R.B. (2008). Axillary cannulation significantly improves survival and neurologic outcome after atherosclerotic aneurysm repair of the aortic root and ascending aorta. Ann. Thorac. Surg..

