# Supplementary material for: Hypothermic Ventricular Fibrillation in Redo Minimally Invasive Mitral Valve Surgery: A Promising Solution for a Surgical Challenge
Source: J Clin Med. 2024 Jul 22;13(14):4269. doi: 10.3390/jcm13144269 (PMC11277611; doi:10.3390/jcm13144269)
Supplement: Supplementary file 1 [file jcm-13-04269-s001.zip › jcm-3071626-supplementary.pdf]

Supplementary Materials

Table S1: Preoperative characteristics

Variables		Study group (n=48)	Control group (n=60)	p-value
Age		70 (65-76)*	60 (50-74)	
Male Gender		31 (65)**	31 (52)	**0.03**
NYHA				0.72
	1	0 (0)	1 (2)	
	2	14 (29)	25 (42)	
	3	27 (56)	27 (45)	
	4	2 (4)	2 (3)	
**Urgency**	Elective	25 (52)	41 (68)	0.12
	Urgent	17 (35)	11 (18)	
	Emergency	6 (13)	7 (12)	
Preoperative renal failure		22 (46)	12 (20)	**0.007**
Extracardiac arteriopathy		13 (27)	2 (3)	**<0.001**
Chronic obstructive lung disease		13 (27)	14 (23)	0.83
Active endocarditis		2 (4)	9 (15)	0.11
Insulin dependent diabetes mellitus		11 (23)	4 (6)	**0.024**
Recent myocardial infarction		1 (2)	0 (0)	0.44
Preoperative stroke		3 (6)	12 (20)	0.05
Preoperative neurologic symptoms		6 (13)	9 (15)	0.90
Pulmonary hypertension		32 (67)	29 (48)	0.10
Coronary artery disease		36 (75)	17 (28)	**<0.001**
Smoking history		12 (25)	17 (28)	0.82
Family history		3 (6)	6 (10)	0.73
Arterial hypertension		45 (94)	40 (67)	**<0.001**
Hyperlipidemia		40 (83)	29 (48)	**<0.001**
Atrial fibrillation		29 (60)	29 (48)	0.33
**Operative indication**				
Mitral valve stenosis		3 (6)	10 (17)	0.14
Mitral valve regurgitation		43 (90)	41 (68)	**0.010**
Endocarditis		2 (4)	9 (15)	**0.011**
Mitral valve prolapse		14 (29)	15 (25)	0.79
Mitral chord rupture		8 (17)	7 (12)	0.64

Anulus dilatation	28 (38)	17 (28)	0.34
Carpentier I	2 (4)	2 (3)	**0.038**
Carpentier II	10 (21)	12 (20)	**0.020**
Carpentier III	20 (42)	11 (18)	**0.012**

NYHA: New York Heart Association; FEV: forced expiratory volume;
*) Continuous variables were described as median and interquartile range
**) Categorical variables were described as mean with the related percentage

Table S2: Intraoperative data

Variables	Study group (n=48)	Control (n=60)	p-value
Surgery time (Minutes)	235 (200-265)*	225 (190-258)	
Time on CPB (Minutes)	154 (228-178)	138 (116-161)	
Mitral valve replacement			
Biological	26 (54)**	22 (37)	0.10
Mechanical	15 (31)	21 (35)	0.83
Mitral valve repair	7 (15)		0.14
Plasty with neochordae	7 (15)	7 (12)	0.87
Number of neochordae	0 (0-0)	0 (0-0)	0.62
Cleft closure	0 (0)	5 (8)	0.064
Segment resection	1 (2)	3 (5)	0.62
Sliding plasty	0 (0)	1 (2)	1
Augmentation	0 (0)	3 (5)	0.25
LAA closure	6 (13)	5 (8)	0.53
Maze procedure	3 (6)	3 (5)	1

CPB: cardiopulmonary bypass; LAA: left atrial appendage
*) Continuous variables were described as median and interquartile range
**) Categorical variables were described as mean with the related percentage

Table S3: Post-operative data

Variables	Study group (n=48)	Control group (n=60)	p-value
Catecholamine duration	22 (17-68)*	18 (12-44)	
Ventilation time (hours)	14 (10-33)	13 (8-19)	
ICU stay (days)	4 (1-6)**	1,5 (1-3)	
Mitral valve re-operation	0 (0)	1 (2)	1
Arrhythmia	10 (21)	5 (8)	0.091
Pneumothorax	3 (6)	2 (3)	0.65
ECMO/right ventricular failure	4 (8)	3 (5)	0.70
Re-thorac. Bleeding	10 (21)	6 (10)	0.19
Renal insufficiency with new onset dialysis	6 (13)	1 (2)	0.042
Stroke	2 (4)	2 (3)	1
Cerebral bleeding	1 (2)	0 (0)	0.44
Periph. vascular complications	0 (0)	1 (2)	1
Sepsis	0 (0)	1 (2)	1
Myocardial infarction	0 (0)	0 (0)	0.25
Pacemaker implantation	7 (14)	1 (2)	**0.021**
30-day mortality	3 (6)	1 (2)	0.32
Intrahospital mortality	3 (6)	1 (2)	0.32

ICU: intensive care unit; ECMO: extracorporeal membrane oxygenation
*) Continuous variables were described as median and interquartile range
**) Categorical variables were described as mean with the related percentage

Table S4: Echocardiographic measurements

Variables	Study group (n=48)	Control group (n=60)	p-value
Pre-OP LVEF	56 (44-62)*	60 (50-65)	
Pre-OP MI II	11 (23)**	11 (18)	0.65
Pre-OP MI III	30 (63)	24 (40)	0.10
Pre-OP MI IV	5 (10)	5 (8)	0.75
Pre-OP MS II	1 (2)	1 (2)	1
Pre-OP MS III	0 (0)	5 (8)	0.06
Post-OP LVEF	55 (44-60)	55 (45-60)	
Post-OP MI I	7 (15)	11 (18)	0.59
Post-OP MI II	0 (0)	2 (3)	0.50
Post-OP MI III	0 (0)	0 (0)	0.27
Post-OP MI IV	0 (0)	0 (0)	0.27
Post OP MS II	0 (0)	0 (0)	0.27
Post OP MS III	0 (0)	0 (0)	0.27

Pre-OP: preoperativ; Post-OP: postoperative; LVEF: left ventricular ejection fraction; MI: mitral valve insufficiency; MS: mitral valve stenosis

*) Continuous variables were described as median and interquartile range

**) Categorical variables were described as mean with the related percentage
